# Supplementary material for: Myc‐dependent endothelial proliferation is controlled by phosphotyrosine 1212 in VEGF receptor‐2
Source: EMBO Rep. 2019 Sep 23;20(11):e47845. doi: 10.15252/embr.201947845 (PMC6832004; doi:10.15252/embr.201947845)
Supplement: Supplementary file 1 — Expanded View Figures PDF [file EMBR-20-e47845-s001.pdf]

Expanded View Figures

Figure EV1. Generation of *Vegfr2$^{Y1212F/Y1212F}$* mice.

A Mice were generated using site-directed mutagenesis on a genomic fragment of the mouse *Vegfr2/Flk1* gene. A substitution mutation, TAT to TTC, was introduced into exon 27 and a mutation-containing targeting vector (red) was transfected into embryonic stem cells, where the vector was inserted into the wild-type (WT) sequence (blue) by homologous recombination. The neo-cassette was removed by crossing with a CAG-Cre-recombinase expressing mouse line, resulting in an 86-base pair remaining insertion into intron 26.

B The correct introduction of the mutation and the otherwise unaffected sequence of the region surrounding exon 27 were verified by Sanger sequencing of forward and reverse strands of DNA from *Vegfr2$^{Y1212F/Y1212F}$* mice, extracted from ear biopsies. The WT and *Vegfr2$^{Y1212F/Y1212F}$ mutant* sequence were aligned. The 1212 codon (blue, tyrosine, wild-type; red, phenylalanine, mutant) and the 86-base pair insertion (yellow) are indicated.

© 2019 The Authors

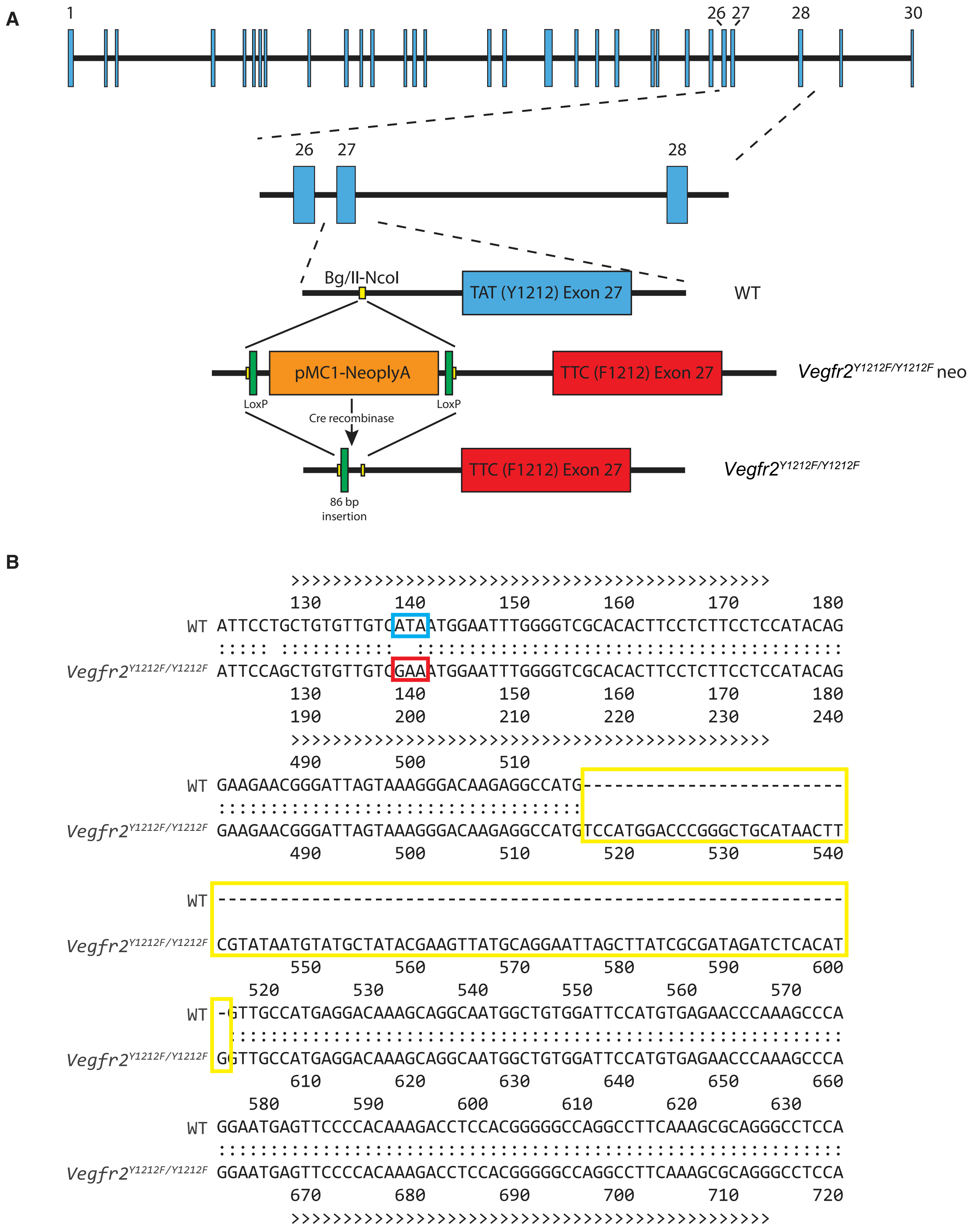

Figure EV1.

 © 2019 The Authors

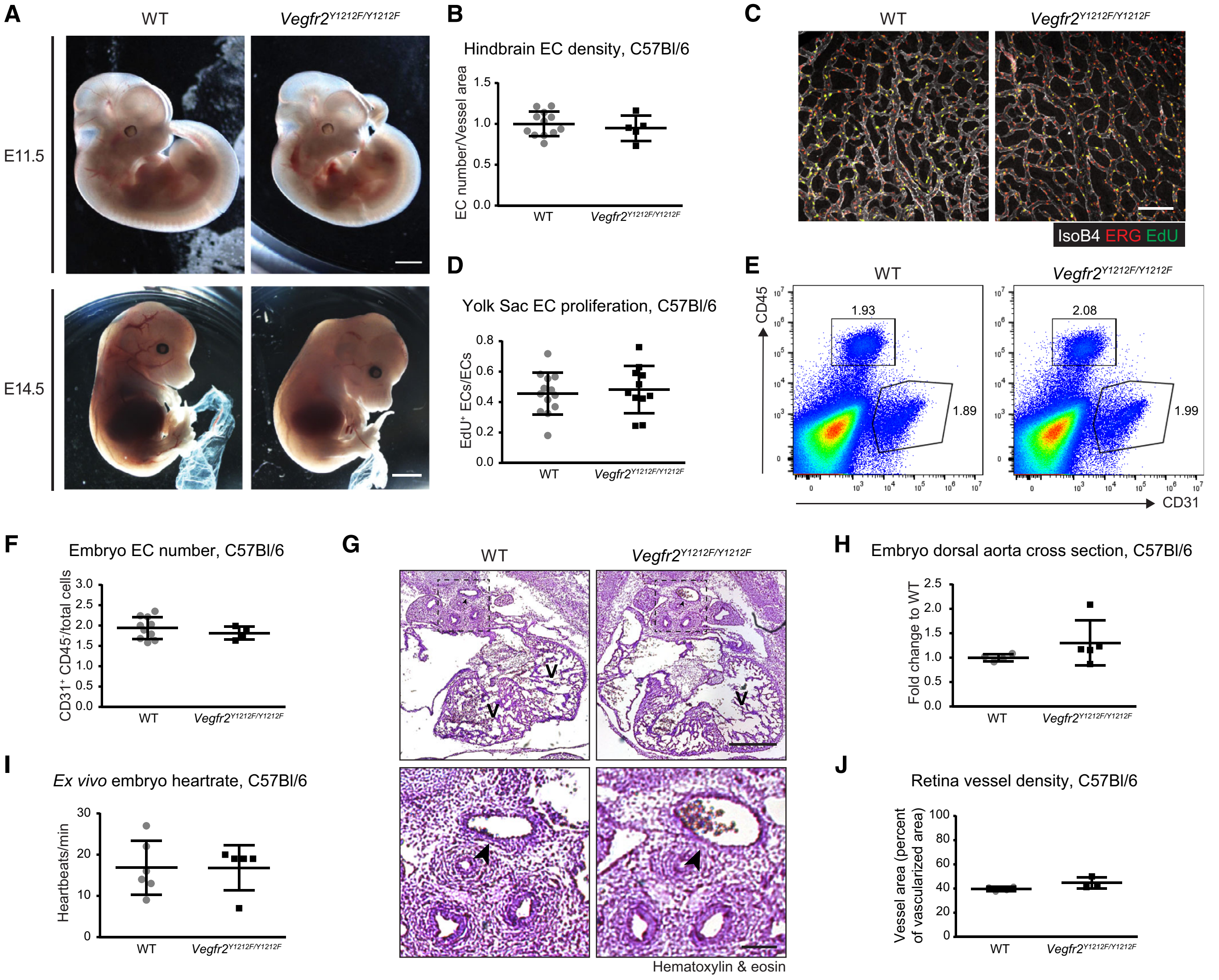

Figure EV2. Embryonic development of C57Bl/6 *Vegfr2*$^{Y1212F/Y1212F}$ mice.

A Embryo morphology. At E11.5 (upper row) and E14.5 (lower row), the C57Bl/6 *Vegfr2*$^{Y1212F/Y1212F}$ embryos showed an overall normal development compared with WT (left). Scale bar, 1 cm.

B EC density in the C57Bl/6 E11.5 hindbrain vasculature. Quantification of ERG-positive ECs normalized to isolectin B4 (IsoB4)-positive area and to WT mean of the litter. Each dot represents the mean of two fields/mouse. Error bars: SD; unpaired *t*-test, not significant. n = 5–12.

C, D EC proliferation in the C57Bl/6 E11.5 yolk sac. (C) Immunostaining for CD31 (white), ERG (red), and EdU incorporation (green). EdU signal was masked with ERG signal. Scale bar, 100 μm. (D) EdU incorporation in ERG-positive ECs (double-positive cells, yellow in C). Each dot represents the mean of three fields/yolk sac, one yolk sac/embryo; error bars: SD. n = 11–13.

E, F EC numbers in the C57Bl/6 E11.5 embryos. (E) Flow cytometry proportion of CD31^{+}/CD45^{-} EC pool in WT and *Vegfr2*$^{Y1212F/Y1212F}$ embryos. (F) Quantification of CD31^{+}/CD45^{-} EC/total cells in C57Bl/6 E11.5 embryos. Error bars: SD. n = 4–11 embryos.

G Heart morphology in the C57Bl/6 E12.5 embryos. V, ventricle; arrowhead, dorsal aorta. Scale bar, 500 μm in upper panels. Magnification in lower panels, scale bar, 100 μm

H Dorsal aorta cross section in the C57Bl/6 E11.5-12.5 embryo hearts. Diameter length expressed as fold change to WT within each litter. Error bars: SD. n = 4–5 embryos.

I *Ex vivo* heart rate in the C57Bl/6 E11.5 embryos. Quantification of the heart rate of explanted C57Bl/6 E11.5 WT and *Vegfr2*$^{Y1212F/Y1212F}$ embryos expressed as heartbeats/min. Error bars: SD. n = 5–6 embryos.

J Vessel density in the C57Bl/6 P6 retina. IsoB4 vessel area normalized to vascularized area in *Vegfr2*$^{Y1212F/Y1212F}$ and WT mice. Error bars: SD. n = 3–4 retinas, one retina/mouse.

© 2019 The Authors

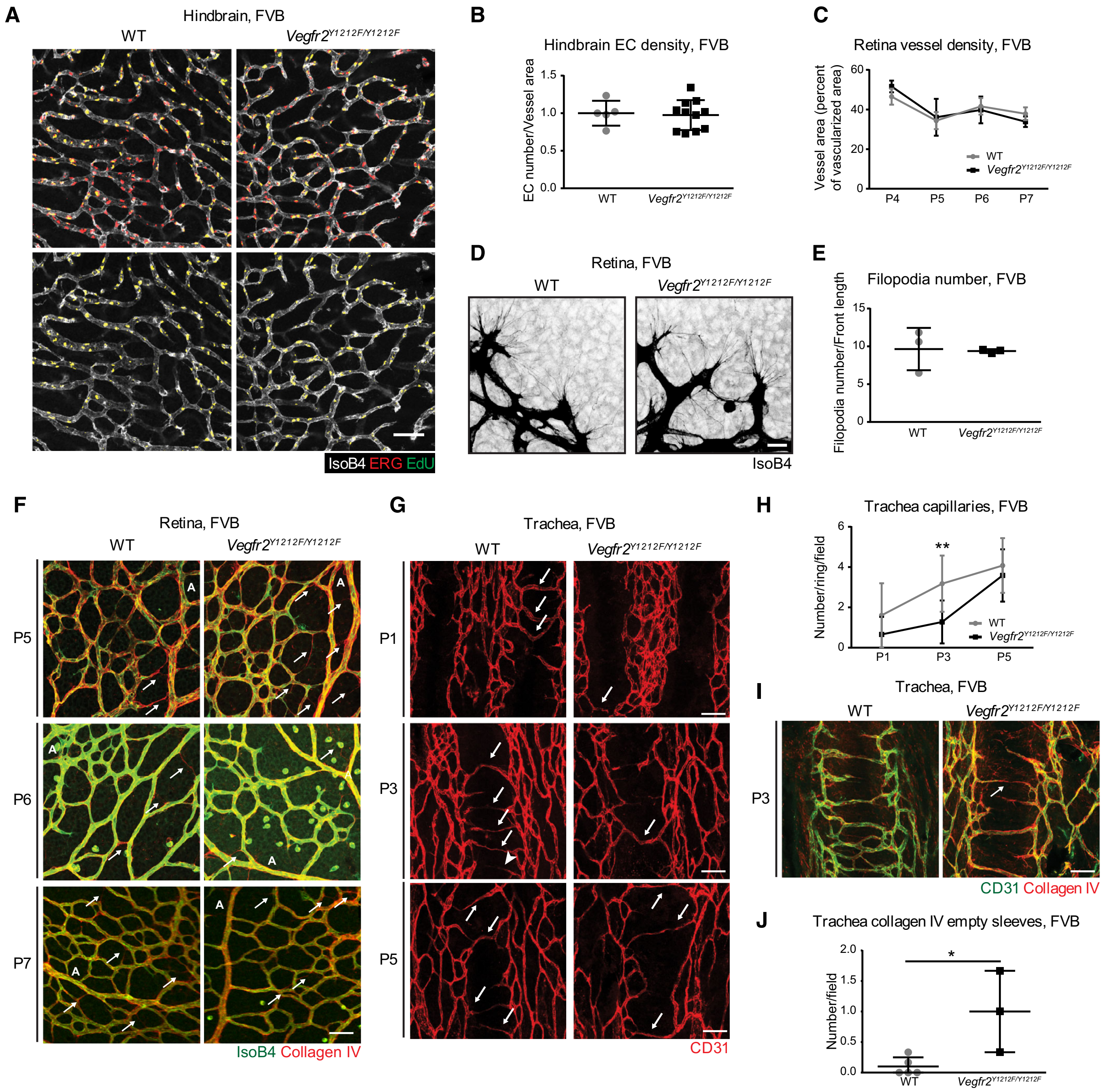

Figure EV3.

© 2019 The Authors

Figure EV3. Postnatal development of FVB *Vegfr2*$^{Y1212F/Y1212F}$ mice.

A EC proliferation in FVB E11.5 hindbrains. EdU incorporation (green) in ERG (red) and isolectin B4 (IsoB4, white)-positive ECs. Double-positive cells (yellow) alone are shown in the lower panel. EdU signal was masked with ERG signal. Scale bar, 50 μm.

B EC density in FVB hindbrain vasculature. Quantification of ERG-positive ECs normalized to IsoB4-positive area and to WT mean of the litter; each dot represents the mean of two fields/mouse. Error bars: SD; unpaired *t*-test, not significant. $n = 5$–11.

C Vessel density in the FVB P6 retina. Isolectin B4 (IsoB4) vessel area normalized to vascularized area. Error bars: SD. P4 $n = 3$–5 retinas, one retina/mouse, P5 $n = 4$–9, P6 $n = 9$–9, P7 $n = 4$–5.

D Filopodia in FVB P6 retina. Isolectin B4 (IsoB4, black)-positive ECs. Scale bar, 20 μm.

E Quantification of filopodia in FVB P6 retina. Number of filopodia by front length in μm. Each dot represents the mean number of filopodia of 3 fields/retina/mouse; error bars: SD. $n = 3$.

F Empty sleeves in the FVB P6 retina. Immunostaining for isolectin B4 (IsoB4, green) and collagen IV (red) in P5-P7 FVB WT and *Vegfr2*$^{Y1212F/Y1212F}$ retinas. Arrows indicate collagen IV-positive empty sleeves. A, artery. Scale bar, 50 μm.

G Postnatal FVB tracheal vasculature. CD31 staining of the capillary bed at P1, P3, and P5. Scale bar, 50 μm. Arrows indicate capillaries spanning the cartilage rings.

H Quantification of capillaries in the FVB trachea. Number of capillaries over one cartilage ring. Error bars: SD; 2-way ANOVA $P < 0.0001$; Bonferroni's multiple comparison test, $^{**}P < 0.01$. P1, 4 rings/mouse; $n = 4$–8. P3, 3 rings/mouse; $n = 3$–5. P5, 3 rings/mouse; $n = 5$–12.

I Collagen empty sleeves in the FVB P3 trachea. Immunostaining for CD31 (green) and collagen IV (red). Arrows show collagen IV empty sleeves. Scale bar, 50 μm.

J Quantification of empty sleeves in the FVB P3 tracheas. Each dot represents the mean number of empty sleeves per field over 3 cartilage rings/mouse; error bars: SD; unpaired *t*-test, $^{*}P < 0.05$; $n = 3$–5.

© 2019 The Authors

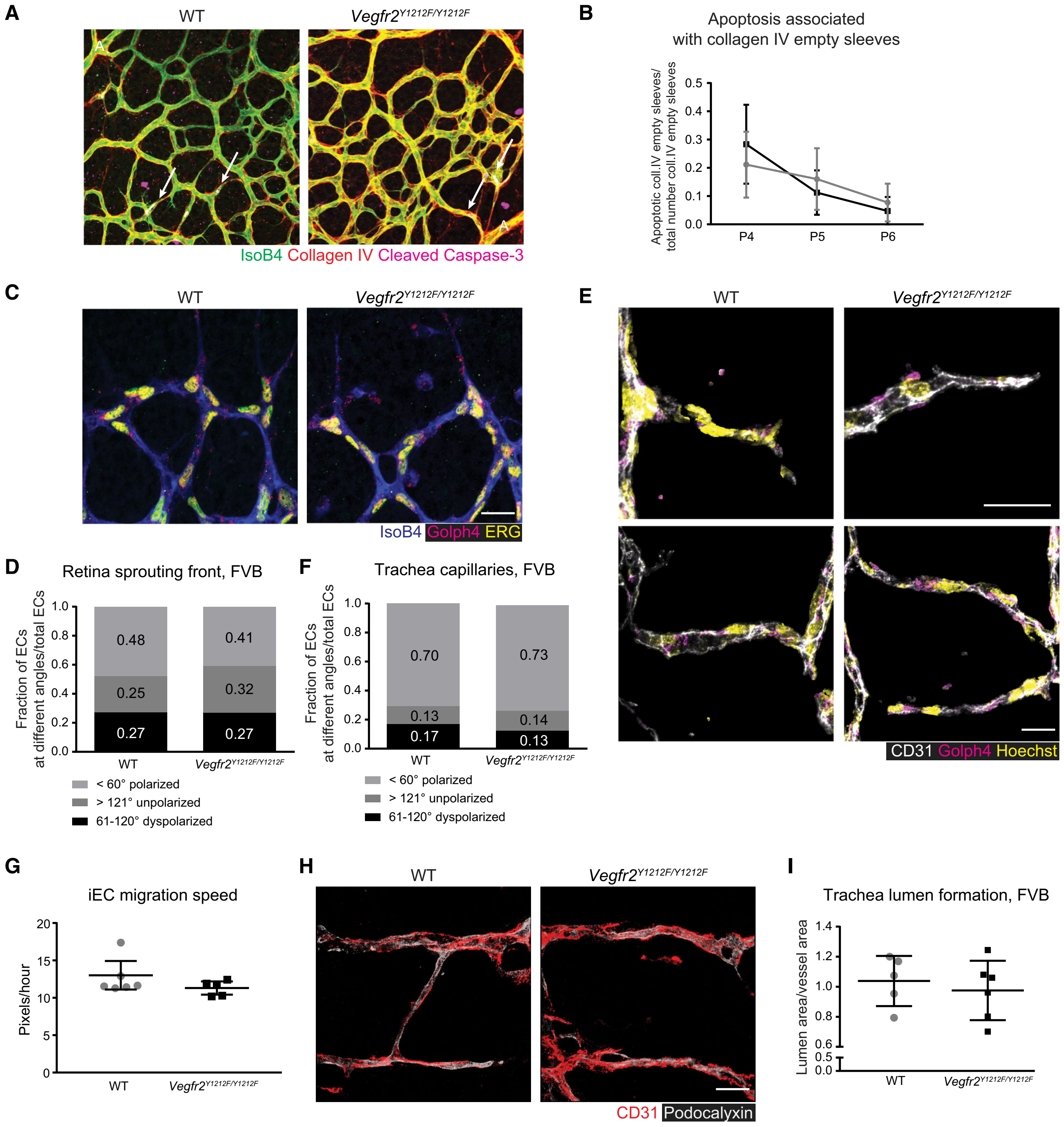

Figure EV4.

 © 2019 The Authors

Figure EV4. pY1212-dependent regulation of apoptosis, polarity, migration, and lumen formation.

A Apoptosis in the developing retina. Immunostaining for isolectin B4 (IsoB4, green), collagen IV (red), and cleaved caspase-3 (pink) in FVB P5 retinas. Arrows, cleaved caspase 3-positive ECs. A, artery. Scale bar, 50 μm.

B Quantification of cleaved caspase-3-positive ECs adjacent to collagen IV empty sleeves, normalized to the total number of collagen IV empty sleeves/field/mouse. Error bars: SD; 2-way ANOVA not significant. P4 n = 6–9 retinas, one retina/mouse, P5 n = 7–12, P6 n = 3–5.

C, D EC polarity in the FVB P5 retina. (C) The angle between the position of the Golgi and the center of the nucleus was determined on retinas immunostained for isolectin B4 (IsoB4, white), ERG (yellow), and Golph4 (magenta). Cells were scored as polarized in the direction of vascular development, i.e., with an angle < 60°, dyspolarized (61–120° angle), or unpolarized (angle > 121°). Scoring was normalized to the total number of ERG-positive ECs. (D) Bar graph shows the ratio of cells scored in the different ranges, < 60°, 61–120°, > 121°. Scale bar, 10 μm; 2-way ANOVA not significant. n = 80–130 cells/retina.

E, F EC polarity in FVB P3 tracheal capillaries. (E) Immunostaining for CD31 (white), nuclei (Hoechst), and Golph4 (magenta). (F) Assessment of Golgi orientation in relation to the nucleus as for (C, D). Number of cells within a certain angle group is normalized to the total number of ECs for each capillary. Scale bar, 10 μm; 2-way ANOVA not significant. n = 12–33 cells/trachea.

G Isolated EC (iEC) migration speed in wound healing assay. Quantification of cell migration speed toward the wound of ECs isolated from wild-type (WT) and *Vegfr2*$^{Y1212F/Y1212F}$ FVB lungs. Each bar represents the mean and standard deviation of the migration speed of each iEC per mouse. Error bars: SD; unpaired t-test, not significant. n = 70–90 cells/2 wells per mouse/5–6 mice/2 experiments.

H, I Lumen formation in FVB P3 tracheal capillaries. (H) Immunostaining for CD31 (red) and podocalyxin (white), in an ImageJ-3D picture reconstruction of trachea capillaries. (I) Quantification of lumen area as podocalyxin-positive area normalized to CD31-positive vessel area. Scale bar, 50 μm; each dot represents the mean of five capillaries/trachea/mouse; error bars: SD; unpaired t-test, not significant. n = 5–6.

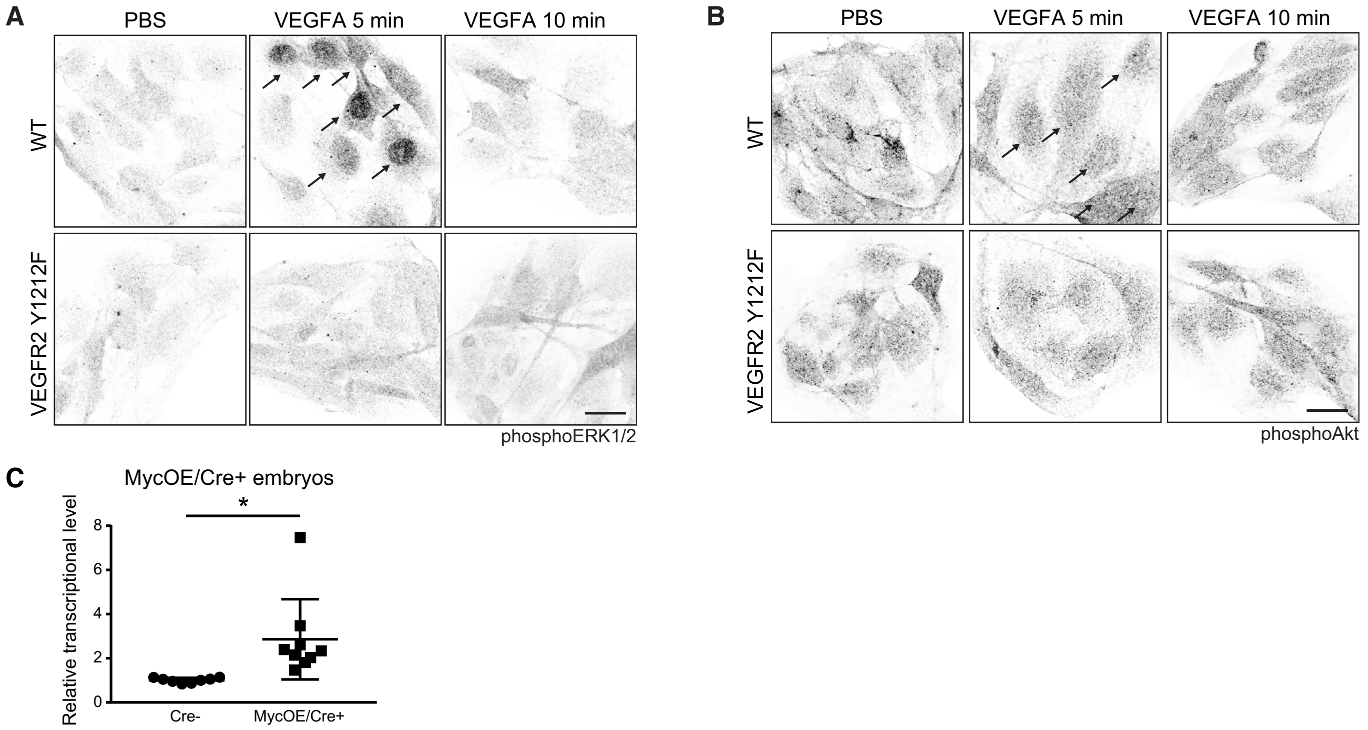

Figure EV5. Induction of pERK1/2, pAkt, and human MYC in WT and VEGFR2 pY1212 conditions.

A phosphoERK1/2 immunostaining (black) of iECs from FVB wild-type (WT) and *Vegfr2*$^{Y1212F/Y1212F}$ lungs. Arrows indicate nuclear accumulation of phosphoERK1/2. Scale bar, 20 μm.

B phosphoAkt immunostaining (black) of iECs from FVB WT and *Vegfr2*$^{Y1212F/Y1212F}$ lungs. Note: Arrows indicate nuclear accumulation of phosphoAkt in response to VEGFA. Scale bar, 20 μm.

C Tamoxifen-induced human Myc gene expression in the E11.5 MycOE/Cre^{+} embryos. Quantitative PCR analysis of human MYC mRNA in E11.5 Myc overexpressing (MycOE/Cre^{+}) and MycOE/Cre^{-} embryos after 100 μl of 4OHT-tamoxifen injection at E9.5. Unpaired t-test, *P < 0.05. n = 8–9 embryos.

© 2019 The Authors
