# Supplementary figures and images for: Myc‐dependent endothelial proliferation is controlled by phosphotyrosine 1212 in VEGF receptor‐2
Source: EMBO Rep. 2019 Sep 23;20(11):e47845. doi: 10.15252/embr.201947845 (PMC6832004; doi:10.15252/embr.201947845)

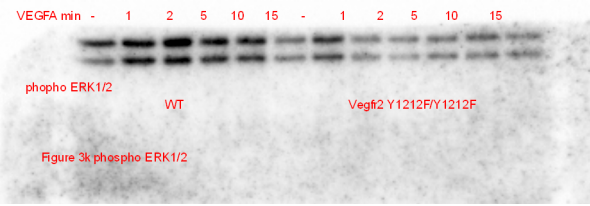

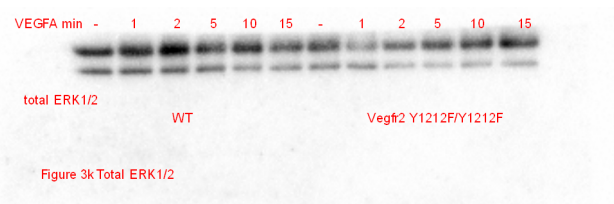

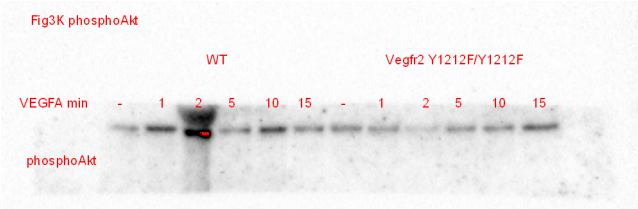

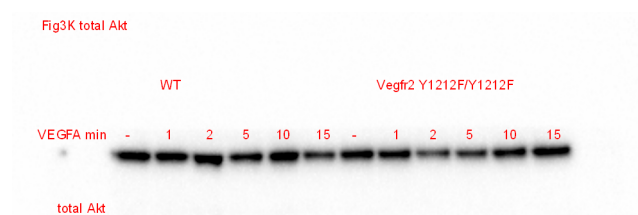

Supplement: Supplementary file 3 — Source Data for Figure 3 [file EMBR-20-e47845-s002.pdf]
